# Effects of N-K management strategies on nutrient uptake efficiency, lodging resistance, and yield in machine-transplanted rice

**DOI:** 10.3389/fpls.2025.1658245

**Published:** 2025-09-08

**Authors:** Zhixin Li, Mingming Hu, Tao Liu, Yuan Tang, Ruhongji Liu, Zhengbo Peng, Cheng Wang, Zhenglan Peng, Zhonglin Wang, Zongkui Chen, Zhiyuan Yang, Yongjian Sun, Jun Ma

**Affiliations:** Rice Research Institute of Sichuan Agricultural University/Crop Ecophysiology and Cultivation Key Laboratory of Sichuan Province, Chengdu, China

**Keywords:** rice, mechanical transplanted, N fertilizer, K fertilizer, nutrient utilization, yield

## Abstract

**Introduction:**

Imbalanced N–K ratios reduce nutrient uptake efficiency while increasing lodging susceptibility, thereby destabilizing yield potential. Optimizing N–K ratios is therefore crucial for improving nutrient efficiency, lodging resistance, and yield potential.

**Methods:**

This study employed the hybrid indica rice cultivar F-you 498 as experimental material. Two K management strategies (basal:panicle = 10:0 and 5:5, denoted as K1 and K2) and three N application regimes (basal:tiller:panicle = 7:3:0, 5:3:2, and 3:3:4, denoted as N1, N2, and N3) were tested. Both fertilizers were applied at identical total rates of 150 kg ha^-^¹ for N and K to investigate N–K interactions on rice growth and nutrient utilization.

**Results:**

N–K interactions significantly affected dry matter accumulation, nutrient uptake, lodging resistance, and yield. Split potassium application (K2) increased grain yield by 3.06% compared with basal-only application (K1), by increasing productive panicles, spikelets per panicle, total spikelets, and seed-setting rate. K2 enhanced post-heading dry matter translocation and improved N–K uptake, elevating panicle N and K accumulation by 5.01% and 13.70%, respectively. K2 also significantly improved lodging resistance. Under K2, the N3 treatment further increased yield by enhancing the number of effective panicles, grains per panicle, and total spikelets, with average yield increases of 12.17% and 4.77% compared with N1 and N2, respectively. Post-heading dry matter accumulation, remobilization ratio, and contribution rate in N3 were higher than in N1 and N2, with two-year average increases of 25.54%, 5.37%, and 7.42% compared with N1, and 12.68%, 2.76%, and 2.57% compared with N2. N3 also promoted the translocation of N and K. Compared with N1 and N2, N3 increased whole-plant N translocation and N transferred to the panicle by 38.09% and 27.45%, and by 14.53% and 12.45%, respectively; whole-plant K translocation and K transferred to the panicle increased by 11.46% and 28.26%, and by 13.35% and 18.35%, respectively. Additionally, N3 improved lodging resistance by thickening internodes and stem-sheath walls. The lodging index was significantly negatively correlated with N and K accumulation in stem-sheaths.

**Discussion:**

Overall, the K2N3 combination enhances post-heading assimilate allocation and nutrient translocation in machine-transplanted rice, strengthens stem mechanical properties, optimizes panicle traits, and ultimately achieves stable and high yields.

## 1 Introduction

As a primary food crop, enhancing the cultivation and productivity of rice has long been a priority in agricultural research. Recent technological advancements and agricultural modernization have amplified the importance of mechanized planting in rice production. Compared with manual transplanting, machine-transplanted rice offers superior operational efficiency and reduced labor intensity (Bhatt et al., 2023), making it a key cultural practice in major rice-growing regions of China. Current research predominantly focuses on planting density (Begum et al., 2022), fertilization methods (Mohapatra et al., 2022), micronutrient supplementation (Jinjala et al., 2024), or single-nutrient management (Jiang et al., 2024). However, persistent misalignment between conventional fertilization practices and the nutrient supply–demand dynamics of mechanized rice systems continues to constrain yield potential. This highlights the urgent need to mitigate structural imbalances in nutrient supply–demand patterns under mechanized cultivation to further unlock rice productivity and safeguard food security.

Nitrogen (N) is one of the essential nutrients required for rice growth. It enhances the synthesis of photosynthates, promotes the differentiation of tillers into productive panicles and florets (Song et al., 2025; Chen et al., 2018), and facilitates N remobilization to grains by regulating the activities of nitrate reductase (NR) and glutamine synthetase (GS) (Wu et al., 2025). An appropriate N application rate (180–240 kg ha^-1^), with a basal-to-panicle fertilizer ratio of 6:4, can help balance source–sink dynamics and improve the efficiency of assimilate translocation. In machine-transplanted rice production, excessive reliance on N fertilizers for yield pursuit, while neglecting balanced potassium (K) application, often results in structural imbalance in fertilization. K plays a critical role in rice growth by acting as an activator of various enzymes, enhancing CO_2_ assimilation, and promoting the translocation of photosynthates to developing grains. Additionally, K regulates N metabolism through its effects on enzymes such as NR and GS, thereby improving N remobilization to the grain and increasing the N harvest index (Gao et al., 2025; Tian et al., 2014). Studies have shown that balanced application of N and K not only improves nutrient use efficiency and reduces nutrient losses that contribute to environmental pollution, but also enhances lodging resistance by promoting lignin biosynthesis and thickening of stem cell walls (Chen et al., 2024; Lei et al., 2025).

In recent years, progress has been made in nutrient management research for machine-transplanted rice. Studies have shown that machine-transplanted rice requires more N (with an optimal N application rate of 225 kg ha^-1^) than conventional cultivation (160–200 kg ha^-1^) (Yao et al., 2021). Therefore, quantifying N demand is crucial for diagnosing N nutritional status and improving N use efficiency in mechanized systems. Further research has revealed that combined N and K application can balance the number of effective panicles and the length of basal internodes in machine-transplanted rice. Specifically, when 180 kg ha^-^¹ of N and 120 kg ha^-^¹ of K were applied, dry matter accumulation and sheath density increased, while bending moment decreased and breaking resistance improved, resulting in significantly higher grain yields (Sun et al., 2017). In addition, increasing K by 30% could offset the adverse effects of a 30% N reduction, significantly improving spikelet number and seed-setting rate, thereby enhancing rice productivity (Deng et al., 2024). However, previous studies have predominantly focused on single-nutrient input strategies or comparisons of fertilization rates, lacking a comprehensive analysis of N–K interactions and their optimal ratios in machine-transplanted rice cultivation. Addressing this gap is essential to resolve the current dilemma in mechanized rice production—high yields without efficiency, and increased output without proportional economic returns. Therefore, this study integrates the agronomic advantages of potted nursery mat transplanting with the physiological characteristics of nutrient demand in rice. The objective is to develop an optimal N–K application pattern that maximizes dry matter accumulation and translocation, nutrient uptake and utilization, lodging resistance, and grain yield. This work aims to provide a comprehensive theoretical foundation and a refined technical framework for achieving high-yield, resource-efficient, and environmentally sustainable rice production under mechanized cultivation systems.

## 2 Materials and methods

### 2.1 Experimental materials

The test variety was F You 498, a hybrid indica rice cultivar with a total growth duration of 155 days. The preceding crop was wheat. Field experiments were conducted during the 2018 and 2019 growing seasons at the Modern Agricultural Science and Technology Park of Sichuan Agricultural University, located in Chongzhou, Chengdu, Sichuan Province (30°42′ N, 103°28′ E). The surface soil layer (0–20 cm) was classified as sandy loam, and baseline soil fertility parameters are shown in **Table 1**.

**Table 1:** Chemical properties of topsoil in the test field from 2018 to 2019.

Year	Organic matter (g kg^-1^)	Total N (mg kg^-1^)	Available P (mg kg^-1^)	Available K (mg kg^-1^)	pH
2018	31.20	1.64	31.71	131.22	6.02
2019	32.31	1.78	32.54	121.67	6.36

**Figure 1:**
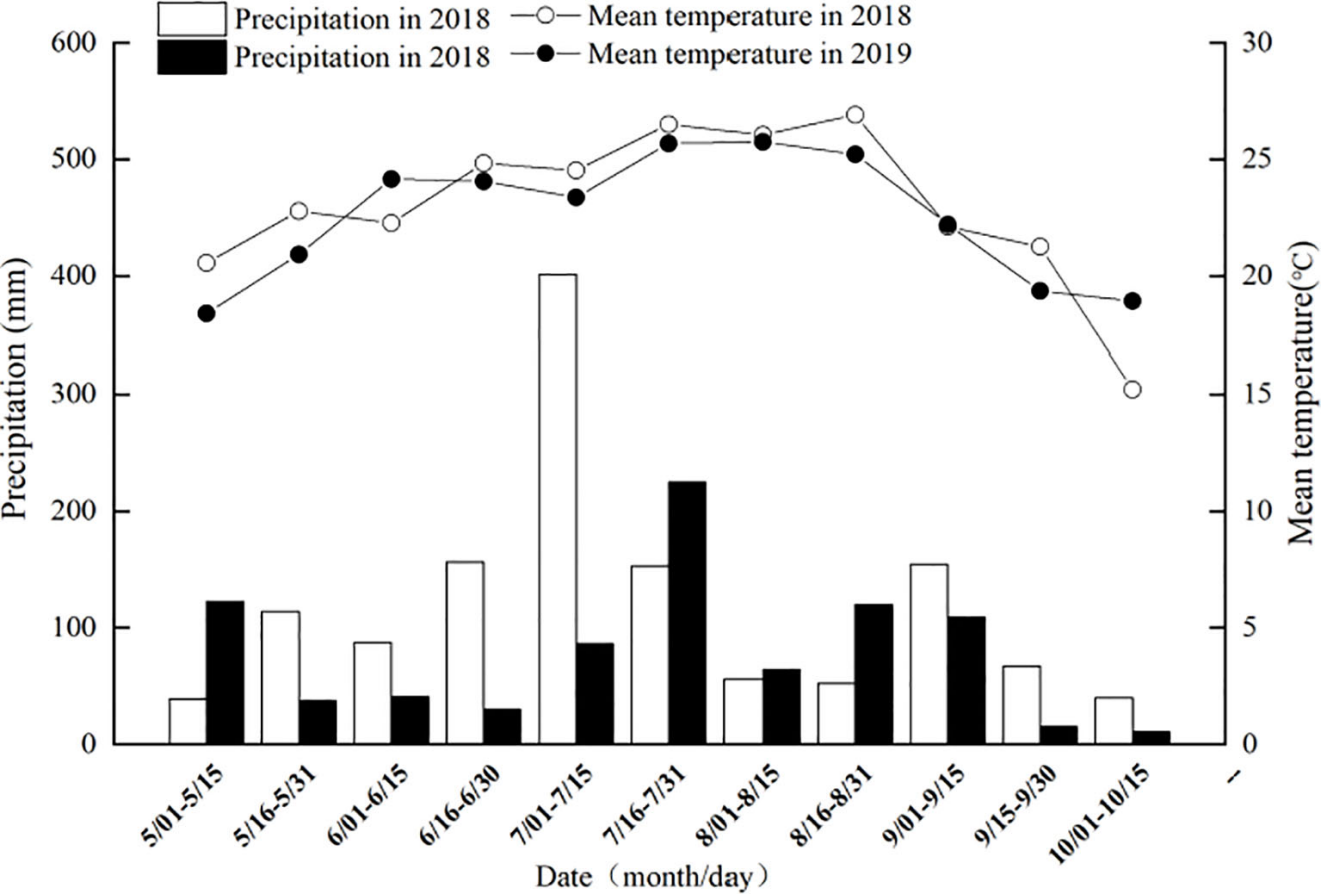
Average temperature and rainfall during the rice growth period in the experimental area from 2018 to 2019.

Meteorological data for the rice growth periods in 2018 and 2019 were obtained from the Sichuan Meteorological Bureau (**Figure 1**).

### 2.2 Experimental design

A split-plot design with two factors was implemented: main plots were assigned to two K application regimes (basal:panicle = 10:0 [K1] and 5:5 [K2]), and subplots to three N splitting regimes (basal:tillering:panicle = 7:3:0 [N1], 5:3:2 [N2], and 3:3:4 [N3]). Fertilizers included urea (N≥46%), calcium superphosphate (P_2_O_5_≥12%), and potassium chloride (K_2_O≥60%). Total N and K inputs were uniformly 150 kg ha^-1^. Basal N and K were applied pre-transplanting, tillering fertilizer at the 5-leaf stage, and panicle fertilizer split equally at the 4th and 2nd leaf tip emergence stages. Phosphorus (P_2_O_5–_75 kg ha^-1^) was broadcast entirely as basal fertilizer.

Pot-mat seedlings were machine-transplanted on May 16, 2018, and May 20, 2019, at a hill spacing of 30 cm × 18 cm with 2–3 seedlings per hill. The experiment comprised 18 plots (three replicates), each covering 15 m^2^. A 30-cm-wide and 20-cm-high soil ridge wrapped in plastic film separated the plots to enable independent water and fertilizer management. All other practices followed conventional field management protocols.

### 2.3 Experimental measurement items and methods

#### 2.3.1 Soil basic fertility determination

Prior to land preparation, soil samples were collected using a five-point sampling method. After natural air-drying and sieving, soil pH, organic matter, total N, available N, available P, and available K were measured using standard analytical methods.

#### 2.3.2 Yield and its components

At maturity, 30 representative hills were selected from each plot to record the number of effective panicles. Five hills per plot were harvested for indoor analysis of spikelet number per panicle, seed-setting rate, and 1,000-grain weight. Grain yield was calculated based on the actual number of hills, excluding border rows.

#### 2.3.3 Aboveground dry matter translocation and accumulation

At rice tillering peak, jointing, heading, and maturity stages, three representative hills from each plot were sampled and separated into stem, leaf, and panicle components. Samples were deactivated at 105°C for 30 min and dried at 80°C to constant weight. The dry weight was recorded and used to calculate the following parameters:


$$\text{Total dry matter}={\text{DW}}_{\text{stem}-\text{sheath}}+{\text{DW}}_{\text{leaf}}+{\text{DW}}_{\text{panicle}}$$

$$\begin{array}{c} \text{Post}-\text{heading dry matter accumulation }({\text{t ha}}^{-1})\\ ={\text{TDM}}_{\text{maturity}}-{\text{TDM}}_{\text{heading}} \end{array}$$

$$\begin{array}{c} \text{Post}-\text{heading dry matter accumulation ratio}(\%)\\ =\frac{\text{PDA}}{{\text{TDM}}_{\text{maturity}}}\times 100 \end{array}$$

$$\begin{array}{c} \text{Contribution rate of post}-\text{heading accumulation}(\%)\\ =\frac{\text{PDA}}{{\text{DM}}_{\text{panicle},\text{ maturity}}}\times 100 \end{array}$$

$$\begin{array}{c} \text{Stem}-\text{leaf dry matter output}({\text{t ha}}^{-1})\\ ={\text{DM}}_{\text{stem-leaf},\text{ heading}}-{\text{DM}}_{\text{stem-leaf},\text{ maturity}} \end{array}$$

$$\begin{array}{c} \text{Output rate of stem}-\text{leaf dry matter}(\%)\\ =\frac{\text{output}}{{\text{DM}}_{\text{stem-leaf},\;\text{heading}}}\times 100 \end{array}$$

$$\begin{array}{c} \text{Transformation rate of stem}-\text{leaf dry matter}(\%)\\ =\frac{\text{output}}{{\text{DM}}_{\text{panicle},\text{ maturity}}}\times 100 \end{array}$$

$$\begin{array}{c} \text{Stem}-\text{sheath dry matter output ratio}(\%)\\ =\frac{{\text{DW}}_{\text{stem}-\text{sheath},\text{ heading}}-{\text{DW}}_{\text{stem}-\text{sheath},\text{ maturity}}}{{\text{DW}}_{\text{stem}-\text{sheath},\text{ heading}}}\times 100 \end{array}$$

$$\begin{array}{c} \text{Stem}-\text{sheath dry matter conversion ratio}(\%)\\ =\frac{{\text{DW}}_{\text{stem}-\text{sheath},\text{ heading}}-{\text{DW}}_{\text{stem-sheath},\text{ maturity}}}{{\text{DW}}_{\text{panicle},\text{ maturity}}}\times 100 \end{array}$$

$$\begin{array}{c} \text{Leaf dry matter output ratio}(\%)\\ =\frac{{\text{DW}}_{\text{leaf},\text{ heading}}-{\text{DW}}_{\text{leaf},\text{ maturity}}}{{\text{DW}}_{\text{leaf},\text{ heading}}}\times 100 \end{array}$$

$$\begin{array}{c} \text{Leaf dry matter conversion ratio}(\%)\\ =\frac{{\text{DW}}_{\text{leaf},\text{ heading}}-{\text{DW}}_{\text{leaf},\text{ maturity}}}{{\text{DW}}_{\text{panicle},\text{ maturity}}}\times 100 \end{array}$$

$$\text{Harvest index}(\%)=\frac{\text{GDM}}{{\text{TDM}}_{\text{maturity}}}\times 100$$

All abbreviations in equations follow conventional agronomic terminology: DW (dry weight), TDM (total dry matter), PDA (post-heading dry matter accumulation), and GDM (grain dry matter) represent biomass measurements. Subscripts specify plant organs (stem–sheath, leaf, panicle) and growth stages (heading, maturity). Units comply with SI standards (t ha^-^¹ for yield, % for ratios).

#### 2.3.4 Nitrogen and potassium uptake and utilization characteristics

Plant samples from Section 1.3.3 were pulverized through an 80-mesh sieve. After wet digestion with concentrated H_2_SO_4_ total N content in organs was determined by micro-Kjeldahl distillation and titration, while total K content was measured via flame photometry. N and K uptake and utilization efficiencies were defined according to Wu et al. (2008) as follows:


$$\begin{array}{c} \text{N}(\text{K})\text{ accumulation}({\text{kg ha}}^{-1})\\ =({\text{DW}}_{\text{stem}-\text{sheath}}\times \text{N}{\left(\text{K}\right)}_{\text{stem}-\text{sheath}})+({\text{DW}}_{\text{leaf}}\times \text{N}{\left(\text{K}\right)}_{\text{leaf}})\\ +({\text{DW}}_{\text{panicle}}\times \text{N}{\left(\text{K}\right)}_{\text{panicle}})\; \end{array}$$

$$\begin{array}{c} \text{N}(\text{K})\;\text{dry matter production efficiency}({\text{kg kg}}^{-1})\\ =\frac{{\text{TDM}}_{\text{maturity}}}{\text{N}(\text{K})\text{ accumulation}} \end{array}$$

$$\text{N}(\text{K})\;\text{grain production efficiency}({\text{kg kg}}^{-1})=\frac{\text{Grain yield}}{\text{N}(\text{K})\text{ accumulation}}$$

$$\text{N}(\text{K})\text{ harvest index}(\%)=\frac{\text{N}{\left(\text{K}\right)}_{\text{panicle},\text{ maturity}}}{\text{N}(\text{K})\text{ accumulation}}\times 100$$

$$\begin{array}{c} \text{Stem}-\text{leaf N}(\text{K})\text{ export amount}({\text{kg ha}}^{-1})\\ =\text{N}{\left(\text{K}\right)}_{\text{Stem}-\text{leaf},\text{ heading}}-\text{N}{\left(\text{K}\right)}_{\text{Stem}-\text{leaf},\text{ maturity}} \end{array}$$

$$\text{Stem}-\text{leaf N}(\text{K})\text{ export rate}(\%)=\frac{\text{Export amount}}{\text{N}{\left(\text{K}\right)}_{\text{Stem-leaf},\text{ heading}}}\times 100$$

$$\begin{array}{c} \text{Contribution rate of stem}-\text{leaf N}(\text{K})\text{ export}(\%)\\ =\frac{\text{Export amount}}{\text{N}{\left(\text{K}\right)}_{\text{panicle},\text{ maturity}}}\times 100 \end{array}$$

$$\begin{array}{c} \text{Increased grain N}(\text{K})\;({\text{kg ha}}^{-1})\\ =\text{N}{\left(\text{K}\right)}_{\text{panicle},\text{ maturity}}-\text{N}{\left(\text{K}\right)}_{\text{panicle},\text{ heading}} \end{array}$$

All equations follow standard agronomic conventions with clear subscript notation: DW (dry weight), TDM (total dry matter), and N(K) (nitrogen/potassium content) are key parameters. Subscripts indicate plant organs (stem–sheath, leaf, panicle) and growth stages (heading, maturity).

#### 2.3.5 Lodging resistance determination

At 20 days post-heading, five uniform plants per plot were selected. For each plant, three stems were measured for internode lengths (S2, S3, S4), fresh weights, and breaking strength of internodes. Breaking force was tested using a 0.001 N digital dynamometer with a 5-cm spacing between support points (Lei et al., 2013; Yang et al., 2013). After testing, stem diameters and wall thickness were measured with a vernier caliper.


Stem diameter (mm)=average of long and short axes

$$\begin{array}{c} \text{Internode resistance}(\text{g cm})\\ =\text{internode}-\text{panicle distance}\times \text{fresh weight} \end{array}$$

$$\text{Breaking resistance }(\text{g cm})=\frac{\text{breaking force}\times \text{support distance}}{4}$$

$$\text{Lodging index }(\%)=\frac{\text{bending moment}}{\text{breaking momen}}\times 100$$

### 2.4 Statistical analysis

Microsoft Excel 2016, SPSS 25.0 (SPSS Institute Inc., Chicago, USA), and Origin Pro 2020 (OriginLab, Northampton, MA, USA) were used for data analysis and visualization. Differences among treatments were tested using the least significant difference method at *P* < 0.05.

## 3 Results and analysis

### 3.1 Yield and its components

Year, K application, and N application rate significantly or highly significantly affected the number of effective panicles, grains per panicle, and grain yield (**Table 2**). The year × K interaction significantly influenced the number of effective panicles, while the year × N interaction significantly affected grains per panicle. The K × N interaction highly significantly impacted the number of effective panicles and grain yield.

**Table 2:** Effects of N-K ratio on yield and yield components of mechanically inserted rice.

Year	Treatment		Effective panicle (m^-2^)	Grains per panicle	1000-grain weight (g)	Total spikelets (× 10^4^ m^-2^)	Seed setting rate (%)	Yield (kg ha^-1^)
2018	K1	N1	160.23c	213.56a	32.18a	3.42b	83.71a	8576.01c
		N2	185.35a	221.71a	32.03a	4.11a	83.75a	9785.06a
		N3	179.69b	224.20a	32.31a	4.03a	79.53b	9117.06b
	Average		175.09B	219.80B	32.17A	3.85A	81.45A	9159.39A
	K2	N1	178.98c	213.34c	32.48a	3.82c	82.86a	8914.89c
		N2	185.70b	226.47b	32.11a	4.20b	81.59a	9465.15b
		N3	192.43a	235.46a	32.62a	4.53a	79.89b	9928.44a
	Average		185.70A	225.09A	32.40A	4.19A	82.33A	9436.16A
2019	K1	N1	198.63c	195.31b	32.12a	3.88c	83.93a	8959.47c
		N2	218.35a	221.25a	32.54a	4.83a	82.54b	10358.98a
		N3	205.87b	222.82a	31.57a	4.59b	80.68c	9897.00b
	Average		207.62A	213.13A	32.11A	4.43A	82.39A	9738.48A
	K2	N1	202.88c	201.80c	32.21a	4.10c	84.23a	9387.57c
		N2	212.08b	217.80b	31.85a	4.62b	82.75b	10129.44b
		N3	218.33a	226.10a	31.88a	4.94a	81.41c	10601.32a
	Average		211.10A	215.23A	31.98A	4.55A	82.80A	10039.44A
One way ANOVA analysis								
Year (Y)			**	**	ns	**	ns	**
K application (K)			**	*	ns	ns	ns	*
N application (N)			**	**	ns	**	**	**
Y×K			*	ns	ns	ns	ns	ns
Y×N			ns	*	ns	ns	ns	ns
K×N			**	ns	ns	ns	ns	**
Y×K×N			ns	ns	ns	ns	ns	ns

Across K treatments, grain yield was higher in K2 than in K1, with an average increase of 3.06% over two years. Under K2, yield increased progressively with panicle N application (N3 > N2 > N1), showing significant differences: N3 increased yield by 12.17% compared with N1 and by 4.77% compared with N2.

Regarding yield components, the number of effective panicles, grains per panicle, total spikelets, and grain-filling percentage were generally higher in K2 than in K1, though most differences were not statistically significant. Under K2, the number of effective panicles, grains per panicle, and total spikelets peaked in N3, significantly exceeding N1 by 7.57%, 11.18%, and 19.57%, and N2 by 3.26%, 3.89%, and 7.37%, respectively. Grain-filling percentage showed a declining trend, with relatively significant differences among treatments.

Different small letters after the data in the same column indicate significant differences between treatments under the same potash treatment and the same year, and the capital letters after the average data indicate significant differences between K1 and K2 (P<0.05) * and ** indicate significant effects at the 0.05 and 0.01 probability levels, respectively, and ns indicates no significant effect.

### 3.2 Dry matter translocation and accumulation

Year and K application highly significantly affected post-heading dry matter translocation (**Table 3**). N application rate and the K × N interaction significantly or highly significantly influenced total dry matter accumulation and post-heading translocation. The three-way interaction (year × K × N) significantly affected the contribution rate of post-heading translocation.

**Table 3:** Effects of N-K management strategies on dry matter accumulation and translocation in machine-transplanted rice.

Year	Treatment		Total dry matter accumulation (t ha^-1^)				Dry matter transport after FHS		
			FTS	JS	FHS	MS	Accumulation (t ha^-1^)	Ratio(%)	Contribution rate (%)
2018	K1	N1	1.22a	2.61b	10.63b	15.40c	4.77b	30.94b	53.90b
		N2	1.31a	2.89a	11.96a	18.04a	6.08a	33.72a	57.83a
		N3	1.13b	2.55b	10.77b	15.70b	4.93b	31.39b	50.38c
	Average		1.22A	2.68A	11.12A	16.38A	5.26B	32.02B	54.04B
	K2	N1	1.10c	2.44b	10.62c	15.97c	5.35c	33.50a	55.64b
		N2	1.27b	2.54ab	11.39b	17.22b	5.83b	33.87a	58.48ab
		N3	1.46a	2.64a	12.18a	18.57a	6.39a	34.44a	60.62a
	Average		1.28A	2.54A	11.40A	17.25A	5.86A	33.94A	58.25A
2019	K1	N1	1.23b	4.15b	13.04c	18.50c	5.55c	30.04c	50.61b
		N2	1.32a	4.38a	15.44a	22.77a	7.33a	32.18a	52.15a
		N3	1.06c	4.00c	13.27b	18.77b	5.84b	31.13b	49.62c
	Average		1.20A	4.18A	13.92A	20.01A	6.24A	31.12B	50.79B
	K2	N1	1.07c	3.88c	12.51c	18.20c	5.69c	31.27c	52.80b
		N2	1.19b	4.01b	13.42b	19.89b	6.47b	32.55b	55.09a
		N3	1.48a	4.22a	14.63a	22.10a	7.47a	33.81a	55.87a
	Average		1.24A	4.04B	13.52A	20.06A	6.55A	32.54A	54.59A
One way ANOVA analysis									
Year (Y)			ns	**	**	**	**	**	**
K application (K)			ns	**	ns	ns	**	**	**
N application (N)			**	*	**	**	**	**	**
Y×K			ns	ns	*	ns	ns	ns	ns
Y×N			ns	ns	ns	ns	ns	ns	ns
K×N			**	**	**	**	**	*	**
Y×K×N			ns	ns	ns	ns	ns	ns	*

FTS, Fully tillering stage; JS, Jointing stage; FHS, Full heading stage; MS, Maturity stages; Different small letters after the data in the same column indicate significant differences between treatments under the same potash treatment and the same year, and the capital letters after the average data indicate significant differences between K1 and K2((P<0.05)) * and ** indicate significant effects at the 0.05 and 0.01 probability levels, respectively, and ns indicates no significant effect.

Across K treatments, total dry matter accumulation at various growth stages mostly showed nonsignificant differences. Post-heading translocation was higher in K2 than in K1, with mostly significant differences among treatments. On average, K2 increased translocation proportion and contribution rate by 5.29% and 7.64%, respectively, compared with K1 over two years. Under K2, total dry matter accumulation at all growth stages peaked in N3, with significant differences observed. At maturity, N3 increased accumulation by 19.02% compared with N1 and by 9.59% compared with N2. Post-heading translocation also peaked in N3, exceeding N1 in translocation amount, proportion, and contribution rate by 25.54%, 5.37%, and 7.42%, and N2 by 12.68%, 2.76%, and 2.57%, respectively.

### 3.3 Assimilate translocation from vegetative organs

Year, N application rate, and the interactions of year with K or N application significantly or highly significantly affected stem–sheath dry matter output rate, stem–sheath dry matter conversion rate, leaf dry matter output rate, and leaf dry matter conversion rate (**Table 4**). K application highly significantly influenced stem–sheath dry matter output rate and stem–sheath dry matter conversion rate. The three-way interaction (year × K × N) highly significantly affected stem–sheath dry matter output rate and stem–sheath dry matter conversion rate, and significantly affected leaf dry matter output rate.

**Table 4:** Effects of N-K management strategies on assimilating translocation from vegetative organs in machine-transplanted rice.

Year	Treatment		SMDOR (%)	SMDCR (%)	LDMOR (%)	Leaf LDMCR (%)	HI (%)
2018	K1	N1	25.07a	16.64a	36.73b	10.22c	59.76b
		N2	18.94b	11.53b	46.21a	12.51b	60.99a
		N3	17.59b	11.35b	49.60a	17.60a	58.42c
	Average		20.54A	13.17A	44.18A	13.44A	59.72A
	K2	N1	20.70a	13.45a	24.64c	7.11c	57.76c
		N2	18.61a	11.79a	36.04b	10.68b	58.71b
		N3	14.29b	8.58b	47.30a	15.11a	59.47a
	Average		17.87B	11.27B	35.99B	10.97B	58.65A
2019	K1	N1	29.94a	20.92a	24.75b	7.42b	58.29b
		N2	31.30a	19.30b	31.83a	9.20a	61.71a
		N3	22.49b	14.53c	29.33a	9.12a	59.08b
	Average		27.91A	18.25A	28.64B	8.58B	59.69A
	K2	N1	27.19a	18.63a	34.58ab	10.06a	57.03c
		N2	22.28b	12.91b	34.44b	10.89a	59.08b
		N3	18.35c	10.66c	38.77a	11.27a	60.51a
	Average		22.61B	14.40B	35.93A	10.74A	58.87A
One way ANOVA analysis							
Year (Y)			**	**	**	**	ns
K application (K)			**	**	ns	ns	ns
N application (N)			**	**	**	**	ns
Y×K			**	**	**	**	ns
Y×N			**	*	**	**	ns
K×N			ns	ns	**	ns	ns
Y×K×N			**	**	*	ns	ns

SMDOR, Stem and sheath dry matter output rate; SMDCR, Stem and sheath dry matter conversion rate; LDMOR, Leaf dry matter output rate; LDMCR, Leaf dry matter conversion rate; HI, Harvest index; Different small letters after the data in the same column indicate significant differences between treatments under the same potash treatment and the same year, and the capital letters after the average data indicate significant differences between K1 and K2(P<0.05) * and ** indicate significant effects at the 0.05 and 0.01 probability levels, respectively, and ns indicates no significant effect.

Across K treatments, stem–sheath dry matter output rate, stem–sheath dry matter conversion rate, and harvest index were higher in K1 than in K2. On average, K1 increased these indices by 19.69%, 22.40%, and 1.61%, respectively, compared with K2 across two years. Under K2, stem–sheath dry matter output rate and conversion rate were highest in N1 (N1 > N2 > N3). Conversely, leaf dry matter output rate, leaf dry matter conversion rate, and harvest index peaked in N3, with mostly significant differences. N3 increased leaf dry matter output rate, leaf dry matter conversion rate, and harvest index by 45.34%, 53.64%, and 4.52% compared with N1, and by 22.12%, 22.30%, and 1.86% compared with N2.

### 3.4 Nitrogen accumulation in organs

Year highly significantly affected N accumulation at the jointing and heading stages (**Table 5**). K application significantly influenced N accumulation at peak tillering, heading, and maturity. N application rate highly significantly affected all indices except N accumulation at peak tillering. The K × N interaction significantly or highly significantly impacted N accumulation at all stages and the N harvest index. The year × N interaction and the three-way interaction (year × K × N) highly significantly affected N accumulation at jointing.

**Table 5:** Effects of different N-K management strategies on nitrogen accumulation in organs of machine-transplanted rice.

Year	Treatment		Stem-sheath				Leaf				Panicle	
			FTS	JS	FHS	MS	FTS	JS	FHS	MS	FHS	MS
2018	K1	N1	10.25a	15.36b	28.03c	19.39c	18.49a	31.68b	51.13b	19.06b	24.09c	96.66c
		N2	9.71b	17.61a	33.50a	24.44a	17.93a	44.24a	53.94a	20.53a	30.30a	119.31a
		N3	9.49b	16.42ab	30.31b	22.60b	16.15b	31.13b	52.29b	19.10b	25.12b	103.67b
	Average		9.82A	16.46B	30.61A	22.14A	17.52B	35.68A	52.45B	19.57B	26.50A	107.55B
	K2	N1	9.20b	16.81b	26.51c	20.20c	16.80c	33.18c	50.02c	19.47c	25.43c	99.75c
		N2	9.31b	17.45b	32.39b	22.20b	18.28b	34.39b	53.39b	21.24b	27.44b	111.81b
		N3	10.79a	19.17a	36.62a	26.18a	21.60a	36.11a	64.59a	23.90a	32.97a	134.30a
	Average		9.77A	17.81A	31.84A	22.86A	18.89A	34.56A	56.00A	21.54A	28.61A	115.29A
2019	K1	N1	9.72a	19.23c	31.90c	21.63c	19.10a	42.81c	53.28c	21.23c	30.79c	96.00c
		N2	9.33b	23.40a	40.93a	25.20a	18.96a	46.48a	58.88b	23.53a	37.94a	123.13a
		N3	9.23b	21.25b	35.22b	23.58b	15.30b	43.90b	67.32a	22.84b	35.70b	118.37b
	Average		9.43A	21.29A	36.02B	23.47B	17.79B	44.40A	59.83B	22.54A	34.81A	112.50A
	K2	N1	8.59c	23.24a	33.20c	22.18c	17.30c	41.72c	53.53c	18.69c	25.74c	98.16c
		N2	9.15b	22.35b	38.71b	26.14b	19.16b	42.83b	65.92b	21.09b	34.28b	116.21b
		N3	11.12a	21.41c	43.67a	29.16a	23.88a	46.90a	71.63a	22.05a	44.22a	129.91a
	Average		9.63A	22.33A	38.19A	25.82A	20.11A	43.82A	63.69A	20.61B	34.75A	114.76A
One way ANOVA analysis												
Year (Y)			ns	**	**	**	*	**	**	**	**	ns
K application (K)			ns	**	**	**	**	ns	**	ns	ns	**
N application (N)			**	**	**	**	*	**	**	**	**	**
Y×K			ns	ns	ns	*	ns	ns	ns	**	ns	*
Y×N			ns	ns	ns	ns	ns	**	**	ns	*	ns
K×N			**	**	**	**	**	**	**	**	**	**
Y×K×N			ns	*	ns	ns	ns	**	**	ns	ns	*

FTS, Fully tillering stage; JS, Jointing stage; FHS, Full heading stage; MS, Maturity stages; Different small letters after the data in the same column indicate significant differences between treatments under the same potash treatment and the same year, and the capital letters after the average data indicate significant differences between K1 and K2((P<0.05)) * and ** indicate significant effects at the 0.05 and 0.01 probability levels, respectively, and ns indicates no significant effect.

Across K treatments, plant N accumulation during growth stages was higher in K2 than in K1, although differences were nonsignificant. At maturity, K2 averaged a 4.11% increase compared with K1. N productivity (dry matter basis) was higher in K1, while the N harvest index was higher in K2, though both differences were nonsignificant. Under K2, N accumulation increased with N application (N3 > N2 > N1), showing significant differences: at heading and maturity, N3 increased accumulation by 36.59% and 31.27% compared with N1, and by 16.16% and 14.69% compared with N2. Grain N productivity decreased with increasing N application (N1 > N2 > N3), with significant differences. The N harvest index peaked in N3, significantly exceeding N1 by 1.70% and N2 by 1.01%.

### 3.5 Nitrogen translocation in vegetative organs

Year highly significantly affected all N export parameters in machine-transplanted rice (**Table 6**). K application, N application rate, and their interaction highly significantly influenced N export amount and increased grain N. The year × K and year × N interactions significantly or highly significantly affected N export amount and the export contribution rate. The three-way interaction (year × K × N) significantly or highly significantly impacted export contribution rate and increased grain N.

**Table 6:** Effects of N-K management strategies on nitrogen translocation in vegetative organs of machine-transplanted rice.

Year	Treatment		Export amount (kg ha^-1^)	Export rate (%)	Contribution rate (%)	Increased grain N (kg ha^-1^)
2018	K1	N1	40.71b	51.41a	42.12a	72.56c
		N2	42.47a	48.57c	35.60c	89.01a
		N3	40.89b	49.51b	38.34b	81.55b
	Average		41.36A	49.83A	38.69A	81.04B
	K2	N1	36.86c	48.17c	36.96a	74.32c
		N2	42.34b	49.35b	37.87a	84.37b
		N3	51.13a	50.51a	38.07a	101.33a
	Average		43.44A	49.34A	37.63A	86.67A
2019	K1	N1	42.31c	49.68c	45.22b	65.21c
		N2	59.52a	54.98a	50.86a	85.19a
		N3	47.69b	50.67b	44.27c	82.66b
	Average		49.84B	51.78B	46.78B	77.69A
	K2	N1	45.86c	52.88b	49.32c	72.41c
		N2	57.40b	54.86a	51.91a	81.93b
		N3	63.10a	55.21a	50.95b	85.68a
	Average		55.46A	54.32A	50.72A	80.01A
One way ANOVA analysis						
Year (Y)			**	**	**	**
K application (K)			**	ns	ns	**
N application (N)			**	ns	ns	**
Y×K			*	*	**	ns
Y×N			**	ns	**	ns
K×N			**	ns	ns	**
Y×K×N			ns	ns	*	**

Different small letters after the data in the same column indicate significant differences between treatments under the same potash treatment and the same year, and the capital letters after the average data indicate significant differences between K1 and K2 (P<0.05) * and ** indicate significant effects at the 0.05 and 0.01 probability levels, respectively, and ns indicates no significant effect.

Across K treatments, most N export parameters were higher in K2 than in K1. On average, N export amount and increased grain N rose by 8.44% and 5.01%, respectively, compared with K1 over two years. Under K2, N export amount, export rate, and increased grain N increased with higher panicle N application, peaking in N3 (N3 > N2 > N1). N3 increased these parameters by 38.09%, 4.62%, and 27.45% compared with N1, and by 14.53%, 1.45%, and 12.45% compared with N2. The N export contribution rate showed inconsistent trends over the years.

### 3.6 Potassium uptake and utilization

Year highly significantly affected total K accumulation, K dry matter production efficiency, and K harvest index at late growth stages (**Table 7**). K application highly significantly influenced total K accumulation at maturity and K harvest index. N application significantly or highly significantly impacted total K accumulation at all stages and K dry matter production efficiency. The year × K and year × N interactions significantly or highly significantly affected the K harvest index. The K × N interaction highly significantly influenced total K accumulation at all stages. The three-way interaction (year × K × N) highly significantly affected K grain production efficiency.

**Table 7:** Effects of N-K management strategies on potassium uptake and utilization in machine-transplanted rice.

Year	Treatment		K accumulation (kg ha^-1^)				K productivity (kg kg^-1^)		KHI(%)
			FTS	JS	FHS	MS	Dry matter	Rice grain	
2018	K1	N1	36.39b	70.06c	162.75c	163.47c	96.02a	59.83a	25.81c
		N2	39.99a	91.09a	191.68a	199.89a	90.27b	52.67b	31.04a
		N3	34.87b	76.52b	174.46b	177.46b	86.59c	49.84c	29.30b
	Average		37.08A	79.22A	176.30A	180.27B	90.96A	54.11A	28.71A
	K2	N1	32.83c	70.56b	164.40c	171.49c	93.34a	56.09a	31.22b
		N2	36.91b	77.20a	179.37b	189.53b	90.87b	52.62b	31.20b
		N3	41.96a	81.52a	194.70a	212.51a	87.39c	49.64c	34.72a
	Average		37.23A	76.76A	179.49A	191.17A	90.53A	52.78A	32.38A
2019	K1	N1	38.30b	85.65c	197.06c	200.30c	77.03a	49.70b	21.21a
		N2	41.90a	104.66a	241.98a	246.37a	70.78c	47.49c	19.14b
		N3	34.75c	87.97b	212.19b	219.43b	72.71b	51.45a	21.27a
	Average		38.32A	92.76A	217.07A	222.03A	73.51A	49.55A	20.54A
	K2	N1	34.39c	81.03c	194.59c	195.37c	76.00a	53.39a	21.71a
	N2	37.37b	95.20b	222.48b	226.62b	71.86b	49.78b	20.21c
	N3	45.48a	104.21a	242.63a	253.17a	68.94c	46.04c	21.23b
	Average		39.08A	93.48A	219.90A	225.05A	72.27A	49.74A	21.05A
One way ANOVA analysis									
Year (Y)			ns	**	**	**	**	**	**
K application (K)			ns	ns	ns	**	ns	ns	**
N application (N)			**	**	**	**	*	**	ns
Y×K			ns	ns	ns	ns	ns	ns	**
Y×N			ns	ns	*	ns	ns	*	*
K×N			**	**	**	**	ns	ns	ns
Y×K×N			ns	**	ns	ns	ns	**	ns

FTS, Fully tillering stage; JS, Jointing stage; FHS, Full heading stage; MS, Maturity stages; Different small letters after the data in the same column indicate significant differences between treatments under the same potash treatment and the same year, and the capital letters after the average data indicate significant differences between K1 and K2((P<0.05)) * and ** indicate significant effects at the 0.05 and 0.01 probability levels, respectively, and ns indicates no significant effect.

Across K treatments, total K accumulation during late growth stages was higher in K2 than in K1, averaging a 3.46% increase at maturity. K dry matter production efficiency was highest in K1, though differences among treatments were nonsignificant. The K harvest index peaked in K2, averaging an 8.49% increase compared with K1. Under K2, total K accumulation peaked in N3, differing significantly from N1 and N2: at heading and maturity, N3 increased accumulation by 21.82% and 26.94% compared with N1, and by 8.83% and 11.90% compared with N2. K dry matter production efficiency peaked in N1 with significant differences, while the K harvest index showed inconsistent trends between years.

### 3.7 Potassium translocation

Year and K application highly significantly affected K export amount, export rate, and increased grain K in machine-transplanted rice (**Table 8**). N application rate, the year × K interaction, the year × N interaction, and the K × N interaction significantly or highly significantly influenced all K export parameters.

**Table 8:** Effects of N-K management strategies on potassium translocation in machine-transplanted rice.

Year	Treatment		Export amount (kg ha^-1^)	Export rate (%)	Contribution rate (%)	Increased grain K (kg ha^-1^)
2018	K1	N1	25.84b	17.56b	61.24a	26.57c
		N2	33.97a	19.77a	54.80b	42.18a
		N3	33.79a	21.21a	65.00a	36.78b
	Average		31.20A	19.51A	60.50A	35.18B
	K2	N1	28.89b	19.66a	53.97a	35.98c
		N2	30.75ab	19.08a	51.98ab	40.92b
		N3	34.28a	19.81a	46.46b	52.09a
	Average		31.31A	19.51A	50.80B	43.00A
2019	K1	N1	25.15a	13.75a	59.22a	28.39b
		N2	25.49a	11.34b	24.05b	29.88a
		N3	22.74b	11.63b	48.73c	29.99a
	Average		24.46B	12.24B	54.00A	29.42A
	K2	N1	28.70a	15.80a	67.68a	29.48b
		N2	25.88b	12.52c	56.49b	30.02b
		N3	29.91a	13.55b	57.67b	31.87a
	Average		28.16A	13.96A	54.62A	30.45A
One way ANOVA analysis						
Year (Y)			**	**	ns	**
K application (K)			**	**	ns	**
N application (N)			**	*	**	**
Y×K			**	**	**	*
Y×N			**	**	**	**
K×N			**	*	**	*
Y×K×N			ns	ns	ns	ns

Different small letters after the data in the same column indicate significant differences between treatments under the same potash treatment and the same year, and the capital letters after the average data indicate significant differences between K1 and K2 (P<0.05) * and ** indicate significant effects at the 0.05 and 0.01 probability levels, respectively, and ns indicates no significant effect.

Across K treatments, K export amount and increased grain K were higher in K2 than in K1, averaging increases of 6.85% and 13.70%, respectively, compared with K1 across two years. Under K2, K export amount and increased grain K increased with higher panicle N application, peaking in N3. N3 increased these parameters by 11.46% and 28.26% compared with N1, and by 13.35% and 18.35% compared with N2. K export rate and contribution rate showed inconsistent trends between years.

### 3.8 Culm physical traits

Year significantly or highly significantly affected internode length, wall thickness, and plant height in machine-transplanted rice (**Table 9**). K application highly significantly influenced the wall thickness of S2 and S3 internodes. N application and the year × K interaction significantly or highly significantly affected wall thickness. The K × N interaction significantly or highly significantly impacted internode length and wall thickness. The three-way interaction (year × K × N) highly significantly affected S4 internode length and the wall thickness of S2 and S4 internodes.

**Table 9:** Effects of N-K management strategies on culm physical traits in machine-transplanted rice.

Year	Treatment		Internode length (cm)			Stem diameter (mm)			Wall thickness (mm)			Plant height (cm)
			S2	S3	S4	S2	S3	S4	S2	S3	S4	
2018	K1	N1	4.80b	7.15c	11.91a	6.96c	6.38b	5.90c	0.83a	0.54a	0.38a	130.63a
		N2	5.71a	8.21a	11.49b	7.31a	6.85a	6.44a	0.75c	0.43c	0.30c	127.40b
		N3	4.38c	7.86b	10.50c	7.18b	6.92a	6.23b	0.78b	0.48b	0.34b	124.17c
	Average		5.02A	7.74A	11.30A	7.15A	6.72A	6.19A	0.79A	0.48A	0.34B	127.4A
	K2	N1	4.74b	8.21a	11.61b	7.30b	6.91c	6.59ab	0.73c	0.50b	0.39b	123.18c
		N2	4.53b	7.70b	11.17c	7.27b	7.04b	6.63a	0.79b	0.57a	0.43a	128.98a
		N3	5.08a	7.31c	12.22a	7.53a	7.25a	6.58b	0.86a	0.43c	0.37b	126.61b
	Average		4.79A	7.74A	11.66A	7.37A	7.01A	6.60A	0.79A	0.50A	0.39A	126.26A
2019	K1	N1	5.55b	7.53c	14.93a	7.29c	6.78c	6.23b	0.68a	0.56a	0.39a	123.59b
		N2	6.86a	8.85a	12.39b	7.60a	7.26a	6.35a	0.44c	0.35c	0.18c	125.59a
		N3	5.06c	8.35b	10.09c	7.36b	7.00b	6.24b	0.58b	0.47b	0.27b	116.26c
	Average		5.82A	8.24A	12.47A	7.42A	7.01A	6.27A	0.57B	0.46A	0.28A	121.85A
	K2	N1	5.64b	8.37a	12.36b	7.39b	6.89b	6.29b	0.51c	0.31b	0.23b	120.65a
	N2	5.52c	8.22b	11.62c	7.47a	7.07a	6.37a	0.65b	0.48a	0.36a	119.22b
	N3	6.21a	8.08c	13.94a	7.44a	6.84c	6.29b	0.69a	0.27c	0.10b	116.67c
	Average		5.79A	8.22A	12.64A	7.43A	6.93A	6.32A	0.62A	0.35B	0.23B	118.85A
One way ANOVA analysis												
Year (Y)			**	*	**	ns	ns	ns	**	**	**	*
K application (K)			ns	ns	ns	ns	ns	ns	**	*	ns	ns
N application (N)			**	ns	**	ns	ns	ns	**	*	**	ns
Y×K			ns	ns	ns	ns	ns	ns	*	**	**	ns
Y×N			ns	ns	**	ns	ns	ns	ns	ns	*	ns
K×N			**	*	**	ns	ns	ns	**	**	**	ns
Y×K×N			ns	ns	**	ns	ns	ns	**	ns	**	ns

S2, penultimate internode from top to bottom of morphology; S3, antepenultimate internode; S4, last four internode; Different small letters after the data in the same column indicate significant differences between treatments under the same potash treatment and the same year, and the capital letters after the average data indicate significant differences between K1 and K2 (P<0.05) * and ** indicate significant effects at the 0.05 and 0.01 probability levels, respectively, and ns indicates no significant effect.

Across K treatments, S2 internode length and S4 stem diameter were higher in K2 than in K1, although differences were nonsignificant; plant height was higher in K1 than in K2, also with nonsignificant differences. Under K2, S2 and S4 internode lengths peaked in N3, while S3 internode length peaked in N1. S2 stem diameter peaked in N3. S2 wall thickness peaked in N3, while S3 and S4 wall thickness peaked in N2, all with significant differences.

### 3.9 Lodging index

Year and K application highly significantly affected internode resistance and breaking moment of all internodes (**Table 10**). N application significantly or highly significantly influenced all parameters except the S4 bending moment. The year × K interaction significantly or highly significantly impacted the S2 and S3 bending moments and the breaking moment. The K × N interaction significantly or highly significantly affected the S2 and S3 bending moments and the S4 breaking moment.

**Table 10:** Effects of N-K management strategies on lodging index in machine-transplanted rice.

Year	Treatment		Internode resistance (g·cm)			Breaking resistance (g·cm)			Lodging index (%)		
			S2	S3	S4	S2	S3	S4	S2	S3	S4
2018	K1	N1	3124.30a	2786.12a	2415.31a	2750.67c	2481.22b	2080.00a	113.59a	112.29a	116.13a
		N2	3003.00b	2701.77b	2375.88b	2771.44b	2486.44b	2038.56b	108.36b	106.39b	116.55a
		N3	2911.87c	2616.23c	2212.00c	2801.56a	2539.78a	1971.89c	103.94c	105.24c	112.19b
	Average		3013.06A	2701.38A	2334.39A	2774.56A	2502.48A	2030.15A	108.63A	107.97A	114.96A
	K2	N1	2999.12b	2631.71b	2113.94b	2656.28b	2355.89b	1801.28c	112.91a	111.73a	117.37a
		N2	2936.17c	2552.94c	2067.86c	2643.11b	2324.78c	1923.33b	111.09b	109.81b	115.26b
		N3	3059.42a	2731.23a	2260.30a	2783.79a	2519.25a	1961.12a	109.90c	108.41c	107.53c
	Average		2998.24A	2638.62A	2147.37A	2694.39A	2399.97A	1895.25B	111.30A	109.98A	113.38A
2019	K1	N1	3669.18a	3398.94a	2986.66a	3193.31b	2834.47c	2388.32c	114.91a	119.92a	125.05a
		N2	3380.82b	3168.31b	2788.35c	3135.60c	2929.42b	2445.58b	107.82b	108.15b	114.02b
		N3	3315.98c	3176.23b	2851.88b	3226.30a	3073.70a	2617.91a	102.79c	103.37c	108.94c
	Average		3455.33A	3247.83A	2875.63A	3185.07A	2945.86A	2483.94A	108.51A	110.48A	116.00A
	K2	N1	3182.19b	2991.65b	2748.84b	2822.56b	2573.70b	2301.59b	112.76a	116.25a	119.43a
		N2	3002.36c	2799.94c	2575.84c	2717.69c	2478.91c	2339.80b	110.48b	112.96b	110.13b
		N3	3340.18a	3070.04a	2797.64a	3079.31a	2770.18a	2637.98a	108.47c	110.83c	106.05c
	Average		3174.91B	2953.88B	2707.44B	2873.19B	2607.60B	2426.46A	110.57A	113.35A	111.87A
One way ANOVA analysis											
Year (Y)			**	**	**	**	**	**	ns	ns	ns
K application (K)			**	**	**	**	**	**	ns	ns	ns
N application (N)			**	*	ns	*	**	**	**	*	**
Y×K			**	*	ns	*	*	ns	ns	ns	ns
Y×N			ns	ns	ns	ns	ns	**	ns	ns	ns
K×N			**	*	ns	ns	ns	*	ns	ns	ns
Y×K×N			ns	ns	ns	ns	ns	ns	ns	ns	ns

S2, penultimate internode from top to bottom of morphology; S3, antepenultimate internode; S4, last four internode; Different small letters after the data in the same column indicate significant differences between treatments under the same potash treatment and the same year, and the capital letters after the average data indicate significant differences between K1 and K2 (P<0.05) * and ** indicate significant effects at the 0.05 and 0.01 probability levels, respectively, and ns indicates no significant effect.

Across K treatments, internode resistance and breaking moment were higher in K1 than in K2. The lodging index of S2 and S3 was higher in K2 than in K1, while that of S4 was higher in K1 than in K2; all differences were nonsignificant. Under K2, internode resistance followed the trend N3 > N1 > N2, with significant differences: N3 increased resistance by 3.53% (S2), 3.16% (S3), and 4.01% (S4) compared with N1, and by 7.76%, 8.38%, and 8.92% compared with N2. The breaking moment of all internodes peaked in N3, with significant differences. The lodging index showed the trend N1 > N2 > N3, with significant differences.

### 3.10 Correlation analysis

Yield showed significant positive correlations with total dry matter accumulation, N accumulation, the N harvest index (NHI), and K accumulation, but significant negative correlations with rice grain N productivity and rice grain K productivity. Both dry matter N productivity and rice grain N productivity, as well as dry matter K productivity and rice grain K productivity, were significantly positively correlated with the S4 lodging index. Additionally, the S2 lodging index was significantly positively correlated with the S3 lodging index.

## 4 Discussion

### 4.1 Effects of N and K ratios on yield and yield components in mechanical transplanting rice

Nitrogen and potassium, as essential macronutrients for rice growth, critically influence yield and its components. Our results demonstrate that under one-time basal potassium application (K1), moderate panicle nitrogen (N2) significantly increased the number of productive panicles and total spikelets by coordinating nutrient allocation between tillering and panicle initiation stages. This effect is attributed to optimized dry matter accumulation from peak tillering to heading under N2, which enhanced photosynthate translocation to panicles (Li et al., 2025). However, split potassium application (K2) altered nitrogen response mechanisms. Under post-anthesis potassium postponement, high panicle nitrogen application (N3) synergistically enhanced yield through three integrated physiological mechanisms: (i) delayed potassium application precisely matched the peak potassium demand during panicle differentiation, activating nitrate reductase and pyruvate kinase activity to promote photoassimilate translocation to panicles, resulting in a 25.54% increase in post-heading dry matter remobilization (Shova et al., 2023); (ii) increased panicle N application delayed root senescence during grain filling and improved N assimilation efficiency, elevating spikelets per panicle by 11.18%; and (iii) split potassium application regulated cell turgor pressure to optimize vascular bundle development, effectively mitigating canopy overcrowding induced by high nitrogen inputs (Islam and Muttaleb, 2016). Insufficient spikelet number constitutes a primary yield-limiting factor in machine-transplanted rice. Potassium supplementation effectively optimizes nutrient supply and physiological conditions during this critical phase, promoting spikelet differentiation while reducing degeneration, thereby significantly increasing grains per panicle (Zhang et al., 2010). Concurrently, enhanced panicle nitrogen synergized with delayed potassium application to boost dry matter accumulation and translocation efficiency during grain filling, ultimately enlarging sink capacity and strengthening source flow for substantial yield improvement.

Regarding yield components under different N–K management strategies, the K2N3 regime exhibited superior performance in productive panicles, spikelets per panicle, and total spikelets. This superiority likely stemmed from improved root uptake of water and nutrients by panicle-stage N–K application, which enhanced tillering capacity and plant vigor. Furthermore, nitrogen application prolonged the active grain-filling phase (Jiang et al., 2022), while potassium regulated filling rate to synergistically increase grain weight (Liu et al., 2024), explaining the yield advantage of K2N3 observed herein. Nevertheless, this combination reduced the seed-setting rate, potentially due to excessive vegetative growth from increased nitrogen proportion at the booting stage, which disrupted nutrient allocation during anthesis and impaired pollination. This observation corroborates the conclusion that N–K imbalance may restrict grain-filling completion through sink–source limitations (Chen et al., 2024). Simultaneously, our analysis demonstrates that optimized N–K stoichiometry significantly enhances economic returns through yield improvement, particularly in the K2N3 treatment, which could effectively increase farmers’ income in local agricultural systems.

### 4.2 Effects of N–K ratios on nutrient uptake and translocation in mechanical transplanting rice

Nitrogen uptake and translocation efficiency constitute the core physiological basis for rice yield formation. Studies indicate that potassium activates nitrate reductase activity, enhancing nitrogen assimilation and partitioning to panicles (Gao et al., 2019) while reducing nitrogen retention in stems and sheaths. Topdressing 50 kg ha^-1^ K at the jointing stage significantly increased plant nitrogen accumulation at flowering, grain nitrogen accumulation at maturity, and the nitrogen translocation contribution rate (Guo et al., 2016). The split application of potassium fertilizer further enhanced nitrogen translocation efficiency by 38.09% through activation of H^+^-ATPase pumps that drive nitrate transporters. The underlying physiological mechanism involves potassium serving as an activator of nitrate reductase, thereby promoting the allocation of nitrogen assimilation products to panicles while reducing nitrogen retention in stems and leaf sheaths (Vijayakumar et al., 2022). When 50 kg ha^-^¹ of potassium was top-dressed at the jointing stage, the treatment significantly increased plant nitrogen accumulation at flowering, grain nitrogen accumulation at maturity, and nitrogen use efficiency (Wang et al., 2025). Concurrently, machine-transplanted rice exhibits significantly elevated nitrogen uptake from heading to maturity. Appropriately increasing the proportion of panicle nitrogen promotes root vitality, facilitating more efficient soil nitrogen absorption and accumulation (Hu et al., 2018; Zheng et al., 2023). Panicle nitrogen application precisely matches the critical period from panicle initiation to grain filling, enhancing shoot nitrogen accumulation and translocation capacity from stems and sheaths to leaves, while increasing nitrogen recovery efficiency by 7.27%–26.06% (Zhang et al., 2022). Thus, split potassium application potentiates nitrogen metabolism, fully unlocking the yield potential of panicle nitrogen to synergistically achieve efficient nitrogen supply for grain sink filling.

The panicle initiation to heading phase represents the peak potassium uptake window, where potassium supply efficacy directly governs subsequent K translocation to grains (Yu et al., 2012). Under the K2N3 regime, potassium accumulation in mature plants increased by 18%–25%. The molecular mechanism involves panicle nitrogen application activating the expression of potassium channel proteins and transporters, which enhances root potassium uptake capacity while optimizing vascular bundle potassium translocation efficiency (Vijayakumar et al., 2024). This finding is corroborated by studies in Iranian rice systems, which demonstrated that in water-scarce regions, application of 120 kg ha^-1^ nitrogen combined with 80 kg ha^-1^ potassium comprehensively improved rice osmoregulation capacity, protected photosynthetic organs, and ultimately enhanced drought resistance while maintaining grain yield (Rabiei et al., 2021). However, excessive potassium application can induce potassium retention in vegetative tissues, inhibiting redistribution to grains. Optimal K application rates have been reported at 108–120 kg ha^-1^ (Hou et al., 2018). Notably, panicle nitrogen systematically enhances fertilizer use efficiency and significantly reduces agricultural greenhouse gas emissions (Poudel et al., 2025) by activating root uptake capacity, strengthening vascular bundle transport efficiency, and optimizing inter-organ allocation strategies. Therefore, by synchronizing nutrient supply with uptake peaks and improving redistribution, K2 synergistically interacts with N3 to systematically enhance productivity, thereby establishing the physiological foundation for efficient potassium accumulation in grains and yield improvement.

### 4.3 Effects of N–K ratios on lodging resistance in mechanical transplanting rice

Machine-transplanted rice exhibits significantly weaker lodging resistance than manually transplanted rice due to inferior stem solidity and root anchorage, necessitating precise N–K co-regulation to synergistically enhance morphological and mechanical traits (Sun et al., 2017). This study is the first to elucidate the mechanism at the level of cell wall construction: under split potassium application (K2), the wall thickness and bending resistance of the S2 internode significantly increased. This improvement is attributed to potassium ions activating the phenylpropanoid pathway, which promotes lignin monomer synthesis and cell wall cross-linking (Ding et al., 2021). Under the K2N3 treatment, the wall thickness and stem diameter of both S2 and S4 internodes increased while shorter basal internodes, directly improving stem bending resistance. At the mechanical level, this combination significantly enhanced bending moment resistance by increasing cellulose deposition and vascular bundle development, thereby minimizing the lodging index. This finding is consistent with Zhang et al. (2021), who reported that “increased potassium application significantly reduces the lodging index by increasing lignin content or cell wall thickness in rice stems.” Moreover, split nitrogen application optimized the enrichment of photosynthetic products and nitrogen in the basal stems, supporting early-stage population establishment while avoiding stem weakness caused by late-stage nitrogen excess, thereby preventing yield losses due to lodging stress (Lu et al., 2025). However, excessive basal nitrogen application (e.g., K1N1) induced gibberellin synthesis, which promoted internode elongation and diluted cell wall material concentration, ultimately reducing mechanical strength.

**Figure 2:**
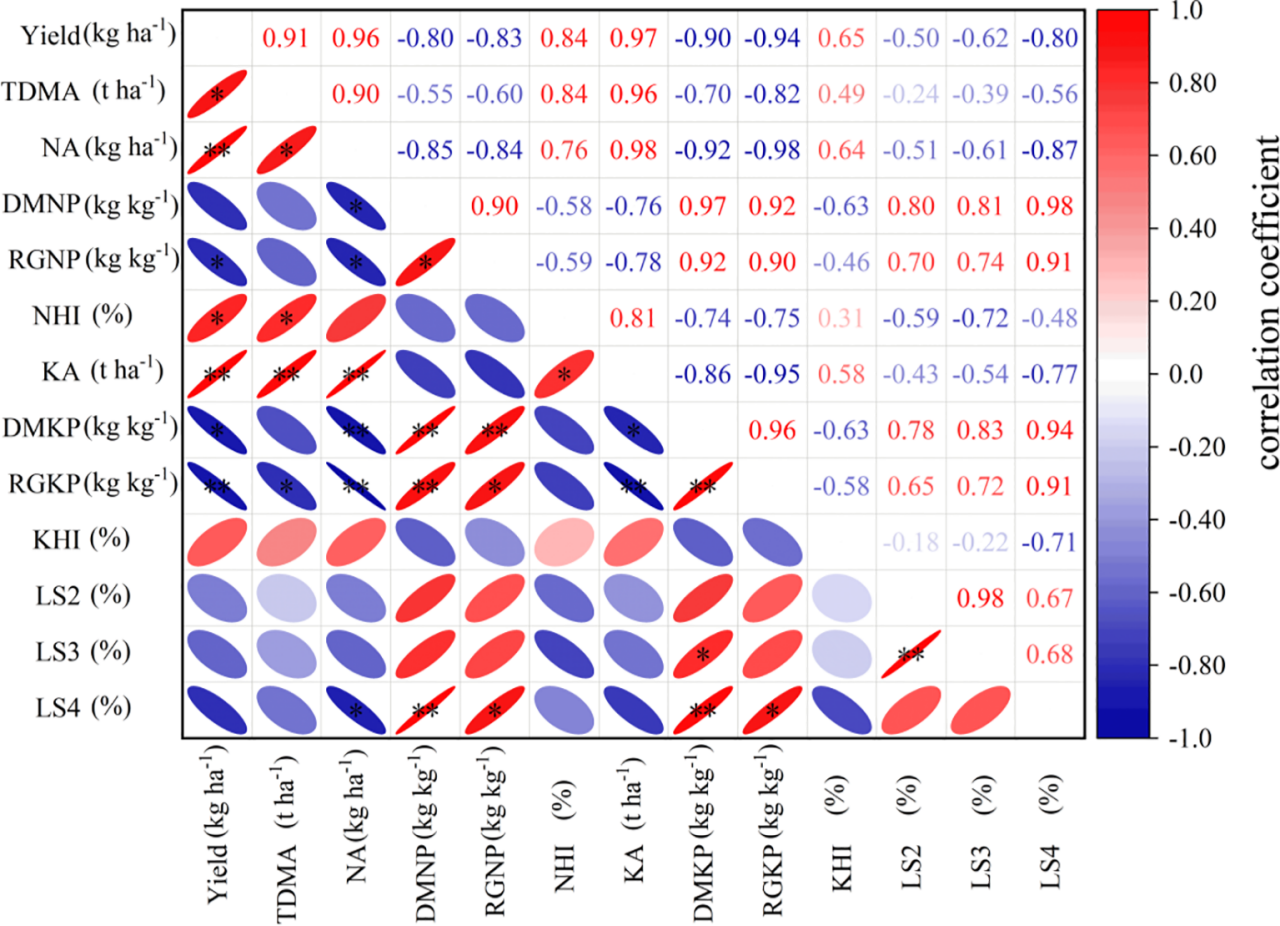
Correlation analysis FHS - Full heading stage, MS - Maturity stages; S2 - penultimate internode from top to bottom of morphology; S3 - antepenultimate internode; S4 – last four internode; Different small letters after the data in the same column indicate significant differences between treatments under the same potash treatment and the same year, and the capital letters after the average data indicate significant differences between K1 and K2 (P<0.05) * and ** indicate significant effects at the 0.05 and 0.01 probability levels, respectively, and ns indicates no significant effect.

As essential macronutrients for plants, adequate nitrogen and potassium directly promote the accumulation of photosynthetic products and assimilate translocation, laying the material foundation for high yields. However, under high-yield conditions, grain output efficiency per unit nutrient decreases, which may stem from the “yield–efficiency trade-off” effect, whereby nutrient use efficiency tends to decline as yields approach higher levels. Regarding stem lodging resistance characteristics, the lodging indices of S2 and S3 internodes showed a significant positive correlation (**Figure 2**), possibly because vascular bundle development in basal internodes is continuous. When the cell wall thickness of the S2 internode decreases, the resulting reduction in bending resistance increases the mechanical load on upper internodes (Yuan et al., 2025). Notably, the dry matter productivity of nitrogen and potassium showed a significant positive correlation with the lodging index of the S4 internode. This result is the first to reveal the intrinsic relationship between nutrient use efficiency and stem mechanical properties. We speculate that the mechanism may involve plants preferentially allocating more resources to grains rather than to the synthesis of stem structural components (e.g., cellulose and lignin) when nitrogen and potassium use efficiency is excessively high, thereby reducing stem mechanical strength. These findings provide new evidence supporting the “yield–lodging resistance synergy” theory (Zhang et al., 2025).

Although this study systematically analyzed the regulatory mechanisms of nitrogen–potassium coordination on yield formation and stress resistance in machine-transplanted rice, some limitations remain. First, the conclusions are based on short-term observations of specific indica rice varieties, and their applicability to japonica ecotypes and long-term cultivation requires further validation. Second, although the improvement in stem lodging resistance is associated with potassium management, key evidence on cell wall component reconstruction (e.g., spatial deposition patterns of lignin) remains insufficient. Third, the environmental risks under high panicle fertilizer application were not assessed. Future research should combine long-term, multi-ecological field trials to deepen the synergistic analysis of post-anthesis nutrient translocation barriers and stem microstructure development, and establish an environmental footprint accounting system to improve the sustainable application of nitrogen–potassium management in machine-transplanted rice.

## 5 Conclusions

Compared with one-time basal potassium application (K1), split potassium application (K2) increased rice yield by optimizing panicle traits, enhancing post-heading dry matter translocation efficiency, and strengthening lodging resistance. When K2 was combined with high panicle nitrogen (N3), the strategy demonstrated amplified effects: it significantly promoted post-heading dry matter accumulation and translocation, improved targeted nitrogen–potassium partitioning to panicles, and elevated grain yield by 4.77%–12.17% compared with conventional nitrogen regimes. Concurrently, this combination reduced lodging risk through increased internode diameter and wall thickness. Collectively, the cultural strategy that integrates delayed potassium with high panicle nitrogen provides an effective agronomic solution for lodging-resistant yield enhancement in machine-transplanted rice by coordinating post-flowering assimilate allocation, strengthening panicle nutrient accumulation, and reinforcing stem mechanical strength.

## Data Availability

The raw data supporting the conclusions of this article will be made available by the authors, without undue reservation.
